# Editorial: The effects of emerging and commonly used medications on the developing brain

**DOI:** 10.3389/fped.2025.1650456

**Published:** 2025-07-08

**Authors:** Jennifer Burnsed, Raul Chavez-Valdez

**Affiliations:** ^1^ Division of Neonatology, Department of Pediatrics, University of Virginia, Charlottesville, VA, United States; ^2^ Division of Neonatology, Department of Pediatrics, The Johns Hopkins University School of Medicine, Baltimore, MD, United States

**Keywords:** developing brain, medications, neonate, neurodevelopment, infant

**Editorial on the Research Topic**
The effects of emerging and commonly used medications on the developing brain

Many medications used in neonates and infants are used “off-label” and their effects on the developing brain are often not well-described. As a necessary part of clinical care, many infants, especially those in the neonatal intensive care unit are commonly exposed to these medications. The neonatal period, particularly in preterm infants, is a time of rapid brain development and is highly vulnerable to the environment and exposures during his critical window. The goal of this Research Topic was to assemble a group of original research papers and reviews on the most up-to-date information and research on effects of commonly used and emerging medications on the vulnerable, developing brain.

In this issue, we present five manuscripts on topics ranging from preclinical models examining the effect of medications on neonatal brain injury to treatment of refractory agitation in neonates to the treatment of infantile spasms in patients with trisomy 21.

Lim et al. published a study on the pharmacokinetics of 7,8-dihydroxyflavone (7,8-DHF) in a neonatal mouse model of hypoxic-ischemic brain injury. This study aimed to test the impact of sex on pharmacokinetics of 7,8 DHF, given that this therapy has shown differing neuroprotection females and males in preclinical models. The pharmacokinetic analysis in this study demonstrates no difference in the pharmacokinetics of 7,8 DHF in males and females but that hypoxic-ischemic brain injury itself was associated with reduced clearance of the drug. These findings will help guide future studies of this promising neuroprotective therapy in neonatal brain injury.

Another preclinical study by Witherspoon et al. in this issue examined the effect of two newer generation anti-seizures medications (ASM) on cell death in the neonatal rodent brain. Several ASMs are associated with neurotoxicity and long-term behavioral changes. This study tested the effects of brivaracetam (BRV) and perampanel (PER) on cell death in the neonatal rat brain and found that neither drug exhibited signs of neurotoxicity. These promising findings suggest that these drugs may offer a safer option for the treatment of early-life seizures.

Chamakioti et al. published a comprehensive review on the role of zinc in the premature brain. The essential micronutrient, zinc, is critical in many facets of neurologic functions and development. Preterm infants are at risk for zinc deficiency and supplementation of zinc is associated with decreased mortality and increased weight gain in this population. This review also highlights some key knowledge gaps in this area and potential future directions in this field of research.

A perspective piece by Beatty et al. discussed refractory agitation in the neonatal intensive care unit population. This paper reviewed high-risk populations and potential therapies and strategies for management of this common problem in hospitalized neonates. This paper highlights a clinical pathway for management of these patients that includes both non-pharmacologic and pharmacologic therapies.

Lastly, Chen et al. utilized the multi-center, prospective National Infantile Spasms Consortium database to examine standard treatments for infantile epileptic spasms syndrome (IESS) in children with trisomy 21. These authors found that epileptic spasms were the first presenting seizure type in children with trisomy 21 and that when compared to vigabatrin and oral corticosteroids, adrenocorticotropic hormone may have superior efficacy for clinical and electrographic outcomes in this population.

This Research Topic issue assembled a wide-ranging collection of papers on the most up-to-date information and research on effects of commonly used and emerging medications on the vulnerable, developing brain.

